# Kriging Surrogate Model for Resonance Frequency Analysis of Dental Implants by a Latin Hypercube-Based Finite Element Method

**DOI:** 10.1155/2019/3768695

**Published:** 2019-04-10

**Authors:** Liu Chu, Jiajia Shi, Eduardo Souza de Cursi

**Affiliations:** ^1^Department of Transportation, Nantong University, Nantong, China; ^2^Département Mécanique, Institut National des Sciences Appliquées de Rouen, Rouen, France

## Abstract

The dental implantation in clinical operations often encounters difficulties and challenges of failure in osseointegration, bone formulation, and remodeling. The resonance frequency (RF) can effectively describe the stability of the implant in physical experiments or numerical simulations. However, the exact relationship between the design variables of dental implants and RF of the system is correlated, complicated, and dependent. In this study, an appropriate mathematical model is proposed to evaluate and predict the implant stability and performance. The model has merits not only in the prediction reliability and accuracy but also in the compatibility and flexibility, in both experimental data and numerical simulation results. The Kriging surrogate model is proposed to present the numerical relationship between RF and material parameters of dental implants. The Latin Hypercube (LH) sampling method as a competent and sophisticated method is applied and combined with the finite element method (FEM). The methods developed in this paper provide helpful guidance for designers and researchers in the implantation design and surgical plans.

## 1. Introduction

The dental implant as a predictable and reliable treatment has been widely applied in the rehabilitation of edentulous patients [1]. The implantation fails sometimes caused by some complicated reasons in oral environment [2]. Fortunately the stability of the implants can be introduced to successfully predict such a failure in most cases by numerical computing or experimental testing [3]. Usually, the stability of implantation can be concluded in two categories: the primary and the secondary stability. The former is largely associated with osseointegration, while the secondary stability is highly corresponding with the bone formulation and remodeling in the process of healing [4–7]. Bone material quality, geometry characteristics of implants, and cortical bone can affect the primary stability [8–12]. The secondary stability obtains from the bone apposition surrounding the interface of implants and bone [13, 14]. The quantification of implant stability offers helpful information to keep reliability in the individual treatment [15, 16].

The histological examination is one of the traditional invasive approaches. Noninvasive methods are required for the observation and measurement of implant stability [17, 18]. The Periotest and the Osstell Mentor system test are two typical noninvasive methods for the measurement of implant stability in diagnosis. In the Periotest, the interfacial damping characteristics between the implant and the surrounding tissue are evaluated [19, 20]. However, the Periotest is often criticized for its lack of prognostic accuracy and poor sensitivity. On the contrary, the Osstell Mentor system is based on resonance frequency analysis (RFA), which appears more competent of assessing the implantation stability [21–25].

The RFA was firstly introduced for dental applications in 1996 and then developed in both experimental and computational aspects [26–31]. It provides an effective way to study the relationship between the stiffness of the implant-bone interface and its surrounding local structures during the healing process [32–34]. In the numerical computation aspect, RFA can be powerfully implemented by a finite element method (FEM). FEM is capable of simulating puzzling geometry characteristics, material properties, and also boundary conditions, which are often tough to deal with in the laboratory [35–37]. Furthermore, it is feasible to allow any independent control of parameters in the implant system by the FEM. Then, performing systematic evaluation for each parameter corresponding to implant stability becomes convenient.

Combining the Latin Hypercube (LH) sampling method with the FEM develops a competent and appropriate stochastic finite element method. The widespread popularity of LH has led to the technical development in various fields, such as the improvement of space filling [38, 39], optimization of projective properties [40–42], minimization of least square error, maximization of entropy [43, 44], and reducing spurious correlations [45]. Meanwhile, LH has been deeply explored in probabilistic analysis, ranging from the assessment of reliability [46–49] to coefficient evaluation for polynomial chaos and merging with the surrogate models [50, 51]. Therefore, the combination of LH and the FEM is a promising method for the RFA of the dental implantation research.

Since the exact relationship between the design variables of dental implants and RF of the system is correlated, complicated, and dependent, traditional regression calculation is difficult to reach a satisfied accuracy. The Kriging model is an interpolation method which finds its roots in geostatistics [52]. As one of the most promising spatial correlation models, the Kriging model is more flexible than the regression model and not as complicated and time-consuming as other metamodels [53]. Recently, there is an increasing interest in applying the Kriging model in the industry, mechanical engineering, and related fields [54–58]. The popularity of Kriging consists in that Kriging is an accurate interpolating approximation model [59]. Kriging model is attractive for its interpolating characteristic, providing predictions with the same values as the observations and reducing the time for the expensive analysis. When there are highly nonlinearity in a great number of factors, polynomial regression modeling becomes insufficient while the Kriging modeling is an alternative choice in spite of the added complexity [60].

The goal of this study is to propose an appropriate mathematical model to evaluate and predict the implant stability and performance. The model is explored to precisely describe the exact relationship between RF and design parameters of dental implants. LH-FEM can reduce the cost of physical experiments, which are expensive and time-consuming. The proposed model in this study has the advantages not only in the prediction reliability and accuracy but also in the compatibility and flexibility in experimental data and numerical simulation results.

This paper consists of four parts.  Section 1 provides a brief overview of the Kriging surrogate model and resonance frequency analysis. Parameters of dental implants are presented in Section 2. Besides, the implementation of LH-FEM is also performed in this section. In Section 3, results are demonstrated and discussed comprehensively. A short summary is concluded in the last section. The methods developed in this paper provide useful information and helpful guidance for implant designers and prosthetic professions in the process of implant design and corresponding surgical plans.

## 2. LH-FEM for Dental Implants

### 2.1. Parameters of Dental Implants

**Figure 1:**
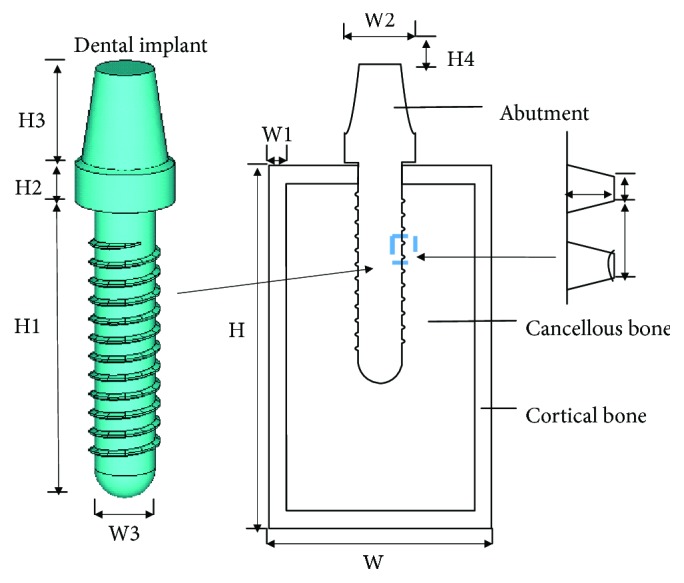
Geometry characteristics of the dental implant.

The geometrical parameters of dental implants are expressed in Figure 1 and Table 1. They include height, length, and width of dental implants, cortical bone, cancellous bone, and ceramic dent. There are 8 parameters (H1, H2, H3, W3, h1, *h*, *w*, and *d*) for dental implants and 2 parameters (W2 and H4) for ceramic dent. *H*, *W*, and W1 for the cortical bone are 26 mm, 16 mm, and 3.1 mm, respectively. With the development of material science, stainless steel, titanium, gold, and even fiber can reach a biomedical grade and can be applied in dental implants. For material properties, Young's modulus, Poisson's ratio, and physical density of dental implants (E1, R1, and D1), ceramic crown (E2, R2, and D2), cortical bone (E3, R3, and D3), and cancellous bone (E4, R4, and D4) are 12 input variables in the finite element model. In order to perform the Latin Hypercube-based finite element method, the specific intervals of parameters corresponding with material properties are given in Table 2.

In terms of the material properties, on the one hand, the work in this study provides the appropriate and wide interval ranges for the material parameters instead of the certain and specific values. The specific values of material parameters are included in the interval ranges. On the other hand, the material properties are analyzed and discussed in the aspects of stiffness and mass matrix, which are more comprehensive to analyze the effects of material properties in RFs of dental implants.

According to the FEM, (1) all materials are homogeneous, isotropic, and linear elastic; (2) the different components exhibit different physical properties; the Young's modulus and Poisson's ratio are given as input variables according to common values in the certain range; (3) perfect bonding is involved between implant-abutment-screw, abutment-crown, and implant-bone. In the contact pair, the objective surface and contact surface are supposed to be bonded. Perfect bonding makes the computation process avoid the problem of convergence, speeds up the calculation, and is proper to have the analysis in large deformation and nonlinear problems; (4) there are no flaws in any components; and (5) for the boundary condition in the FEM of the dental implant system, three surfaces (in the bottom and left and right sides) of the cortical bone surface are chosen and completely fixed; the displacement in X, Y, and Z directions of these three surfaces is constrained to be zero.

Besides, in the previous work of Chu et al. in reference [49], it has been proven that a sufficient number of samples can guarantee a satisfied accuracy of LH and MCS. Enlarging the sampling pool can effectively improve the result accuracy when the number of samples is small; however, when the amount of samples reaches a certain number, the improvement is not evident. Therefore, the number of samples for each variable is supposed to be 500 to obtain an appropriate accuracy while reducing computation costs.

### 2.2. Latin Hypercube-Based Finite Element Method

**Figure 2:**
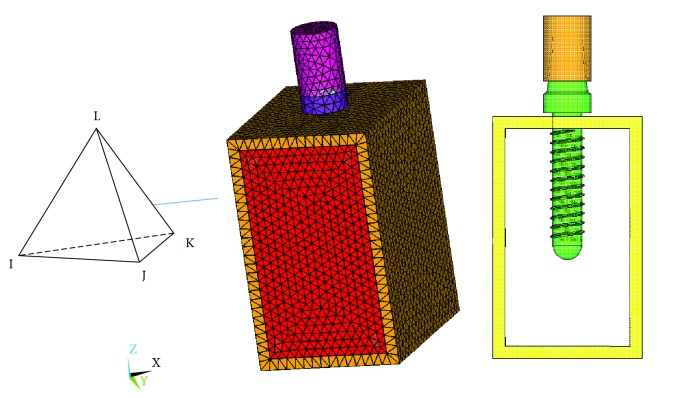
Finite element model of dental implants.

A finite element model of the dental implant system is created as shown in Figure 2 by ANSYS (Mechanical Parameter Design Language, Version 14.5, USA). Solid 285 is the chosen finite element, which is a tetrahedron solid element with 4 nodes in each element, and each node has 3 degrees of freedom (displacement in X, Y, and Z directions). The tetrahedron shape of Solid 285 is flexible and convenient to mesh nonlinear and complicated geometry components, such as thread of dental implants. There are a total of 133961 elements and 22168 nodes. The thread components of dental implants have been fine meshed. By performing a finite element (FE) procedure, the RF can be obtained by solving the eigenvalue problem in the govern equation through the block Lanczos method.

The study of the relationship between the various corresponding parameters of the dental implant system and the values of RF requires fabrication of a huge sample set for experiments, which is expensive and time-consuming. LH as an advanced Monte Carlo method is especially efficient, which divides the sample space into a number of subspaces, then samples from subspaces, thereby perfectly avoiding sample clustering or variation in the boundary [49, 50]. Combing the traditional finite element model of dental implants with LH is a sophisticated method to overcome the disadvantages of physical experiments.

**Figure 3:**
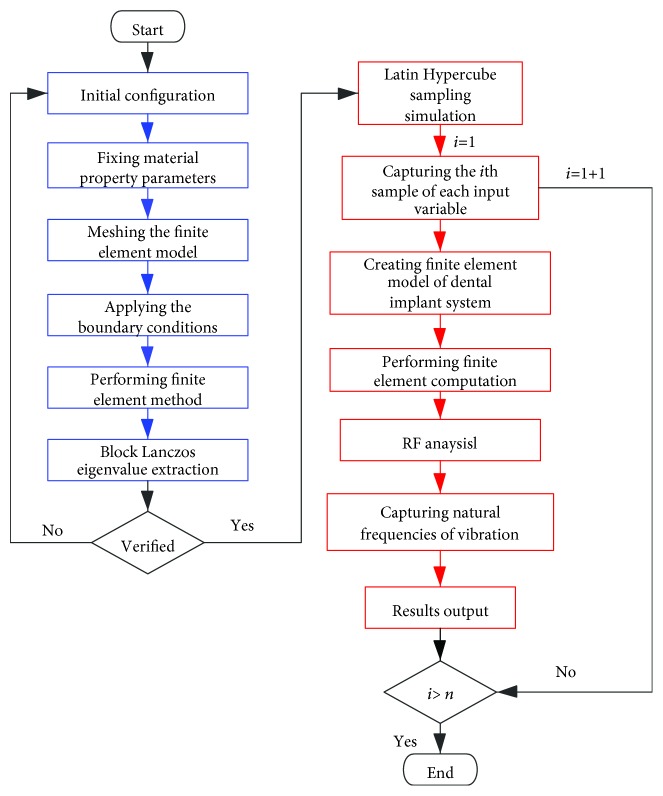
The flowchart of LH-FEM.

The input variables of the finite element model were assigned using LH sampling to effectively avoid sample repetition or clustering. For each variable, there are five hundred randomly samples, which are uniformly distributed in the specific interval range as shown in Table 2. The stochastic sampling process of the input variable creates a reliable database for FEM computation. The flowchart of LH-FEM in Figure 3 presents the programing process. The process marked by blue color represents the deterministic finite element model of the dental implant system. The convergence and accuracy of the deterministic finite element model for dental implants are verified before the next steps. After the validation of the deterministic finite element model, the original codes are assigned to LH-FEM. The loop of LH-FEM does not stop until all of the samples are computed by the finite element model. Then, the results of RF are captured and stored in the output database, as shown in the right side of the flowchart in Figure 3 which are marked by red color.

**Figure 4:**
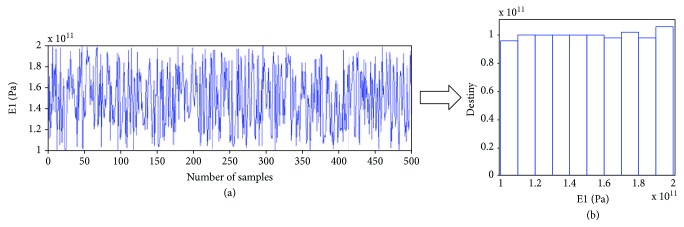
Sample records of Young's modulus of dental implants by LH ((a) for stochastic sampling record and (b) for probability density distribution result, respectively).

In order to find the availability of LH-FEM, a parameter sampling record (E1) of input variables is presented in Figure 4. The difference between samples is not regular because of the stochastic sampling process, which makes the sampling set including different situations as far as possible. The probability distribution in statistical mathematics is following a uniform distribution as in Figure 4. The LH sampling method is successfully implemented in the whole sampling spaces.

## 3. Results and Discussion

### 3.1. Results of the Deterministic Finite Element Model

**Figure 5:**
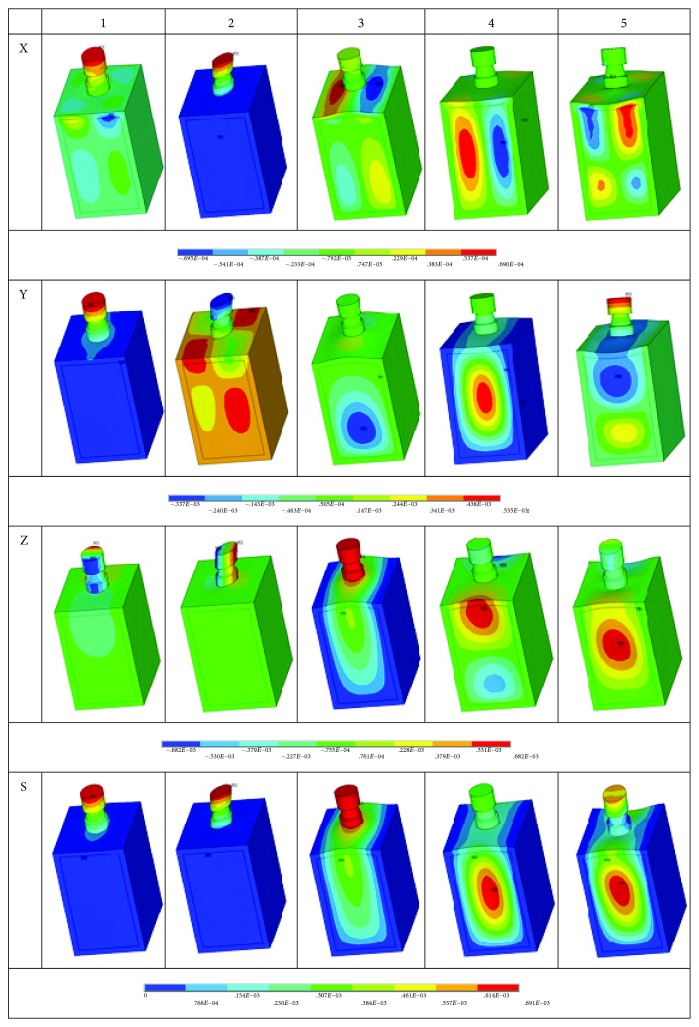
Displacement in the first five order RFA (X, Y, and Z axis directions and sum vector).

RFA (resonance frequency analysis) as a noninvasive and nondestructive technique is proposed to assess the implant stability. A deterministic finite element model of dental implants is performed to evaluate the feasibility of this method. The resonant displacements of the first five order resonance frequencies are plotted in Figure 5, for the X, Y, and Z directions and vector sums, respectively. The axes in Figure 5 are the same as that in Figure 2. The contour results allow visualizing the displacement and mode shape of the dental implant system. The contours present that the displacement happened in the direction of Y axis in RFA is a more dominant vector that influences the whole system in the first-order RFA than others. While in the second-order RFA, the more important displacement is observed in the direction of X axis. In addition, the displacement vector sums in the third-order RFA are more approximated to the displacement in the direction of Z axis. For the fourth- and fifth- order RFA, the displacements in vibration modes are more complicated and the natural frequencies are larger than the lower-order vibration modes. Besides, the largest deformation in the first- and second-order vibration modes happens in the top of the system, which well agreed with the results in the work of Li et al. [36].

**Figure 6:**
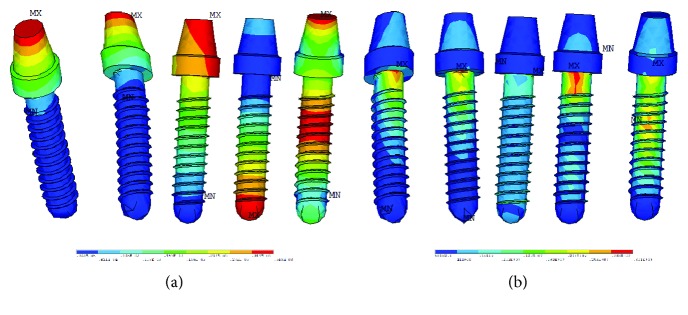
Results of dental implants in the first five order RFA ((a) displacement vector sums, (b) Von Mises stress of the dental implant).

In order to be more convenient, the results of dental implants are demonstrated independently.  Figure 6 presents the displacement vector sum and Von Mises stress of dental implants in the first five order RFA. The associated displacement corresponding to the first bending modes of a cantilever beam has been experimentally observed in the literature [61]. The results of the deterministic finite element model are consistent with those in experiments of the literature. The accordance can be confirmed in both the displacement vector sums and Von Mises stress. The failures and risks most likely occur at the beginning of the threads and the junction of implant collar. This fact is in a good agreement with numerical and experimental investigation [37, 62]. Therefore, the results in this study are validated. The finite element model of dental implants is feasible and appropriate for further studies.

In this study, not only the resonant frequencies of dental implants are calculated by the FEM but also the vibration modes of the dental implants are provided. As the direct measurement of the vibration performance of dental implants higher than the third mode is difficult, the displacements of dental implants under the vibration mode in this numerical study are an important supplement of the experimental measurements. Furthermore, the high mode vibration is an appropriate reference for the safety and reliability of the dental implants in the real operating environment.

### 3.2. Probabilistic Results of LH-FEM

The results of LH-FEM are a large database, and the effective and useful information is extracted and presented in Table 3. The maximum, minimum, mean value, and variance of the first five order RFs are all shown in Table 3. The results of the first-order and second-order RFs are very close, which is because of the geometrical symmetry in the finite element model. However, the vibration modes of the first and second orders are different in Figure 5. In the first-order vibration mode, the dominant displacement happens in the direction of Y axis, while in the second-order vibration mode, the important displacement occurs in the direction of Z axis.

**Figure 7:**
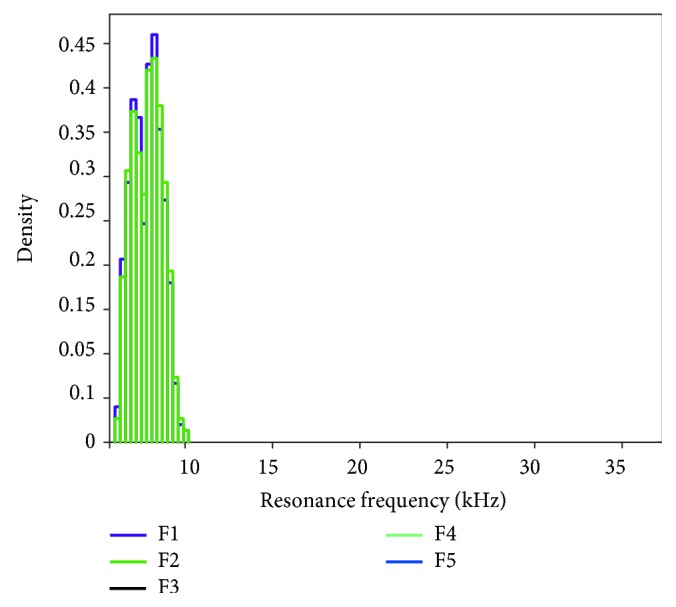
Probability density of the first five order RFs.

**Figure 8:**
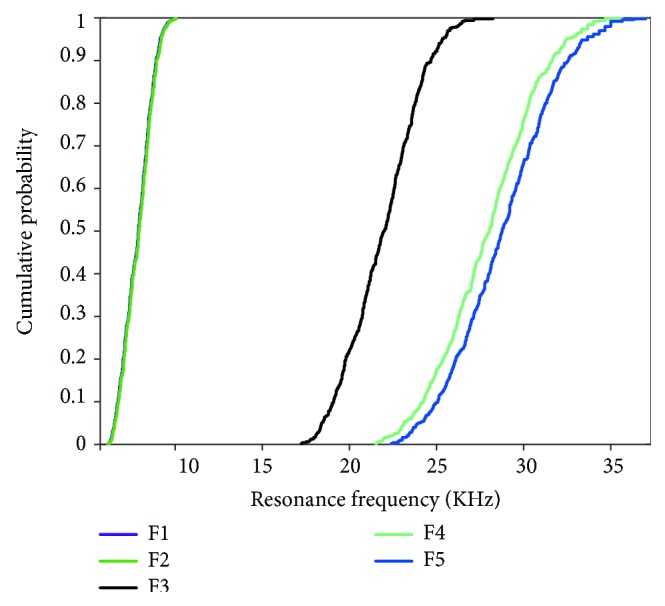
Cumulative probability of the first five order RFs.

Different with the probability distribution of input variables, the output results of the first five order RFs for dental implants do not have uniform distributions as shown in Figures 7 and 8. From Figure 7, the results of first- and second-order RFs are more concentrated in the smaller interval than the third, fourth, and fifth orders, which means the difference in material property of dental implants causes a large deviation of RF in higher-order vibration modes. Besides, in Figures 7 and 8, the probability distribution and cumulative probability of the first- and second-order RFs are approximated, but when compared with the third-order RF, the difference is very evident. These characteristics of the probabilistic results provide helpful guidance in experimental designs and research.

Furthermore, the primary (first order) RF of LH-FEM is fluctuated in the interval range from 6100 to 10000 Hz approximately, and the average is 7800 Hz. In regard to RFA, tremendous substantial studies have been accomplished in experiments and clinical research. In the work of Glauser et al. [26], the implant stability based on the RFA of dental implants under an early functional loading is explored. During a 12-month interval, the average RF ranges from 6100 Hz at the 1st month to approximately 6600 Hz at the 12th month. Besides, the results of the experiment in the tibia of three groups of guinea pigs in a period of 4 weeks [34] also indicated that the average RF is roughly 5650 Hz amongst the three groups. Compared with these literature data, the results of LH-FEM are in a reasonable range. Therefore, the database of LH-FEM for RFA of dental implants is available and feasible.

The work of this study provides the maximum, minimum, mean values, and the variances of the resonant frequencies of dental implants. The primary (first order) resonant frequencies of dental implants corresponding to the implant stability in the literatures fall within the interval range of LH-FEM, which strongly proves the feasibility of the proposed model in this study. Besides, the probability density distribution results of the first five order RFs for dental implants in Figures 7 and 8 provide important information corresponding to the safety and reliability of dental implants' stability.

### 3.3. Prediction of the Kriging Surrogate Model

The resonance frequency is directly attributed to the stiffness matrix and mass matrix. The material stiffness and the stiffness of implant-bone interfaces and surrounding tissues have positive effects on RF, while RF usually has the inverse proportion to the mass matrix. However, the Young's modulus of the cancellous bone is crucially related with the physical density, and the increase of physical density of cancellous bones (mass matrix) can contribute to the improvement of the Young's modulus (stiffness matrix). On the other hand, cancellous bones have a much larger contact area to the dental implant and can hugely affect the whole dental implant system. Therefore, the relationship between RF and parameters of material properties in the dental implant system is complicated and correlated. It is difficult to be described by a simple linear interpolation or regression function.

**Figure 9:**
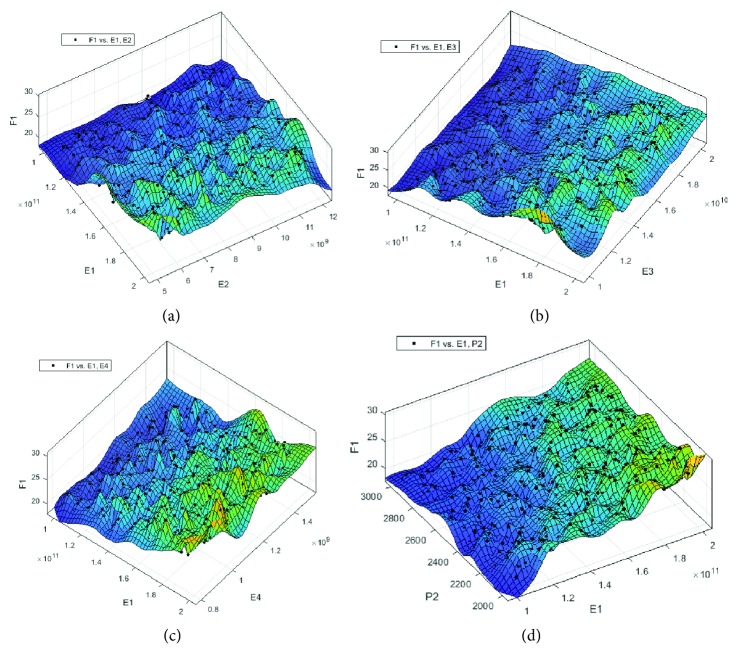
Prediction results of the Kriging surrogate model ((a), (b), (c), and (d) represent the relationships between E1, E2, and F1; E1, E3, and F1; E1, E4, and F1; and E1, P2, and F1, respectively).

Based on the reliable database of LH-FEM, a huge amount of samples for parameters of material properties in the dental implant system and corresponding RF is provided. By performing the Kriging surrogate model, the numerical relationship between RF and parameters of material property is created. The accuracy and convergence of the prediction by the Kriging surrogate model are demonstrated in Figure 9. The black spots in Figure 9 are prediction results of the Kriging surrogate model; the mesh surface is the results obtained from LH-FEM. Satisfied level of accuracy is reached by the Kriging surrogate model. Figure 9 well confirms the prediction accuracy of the proposed Kriging surrogate model in this study.

**Figure 10:**
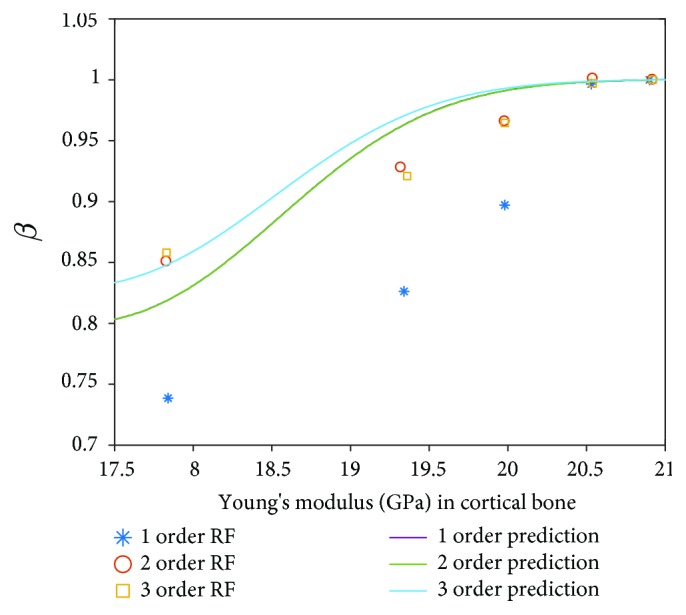
Comparison between deterministic results and prediction results of RF in the cortical bone.

**Figure 11:**
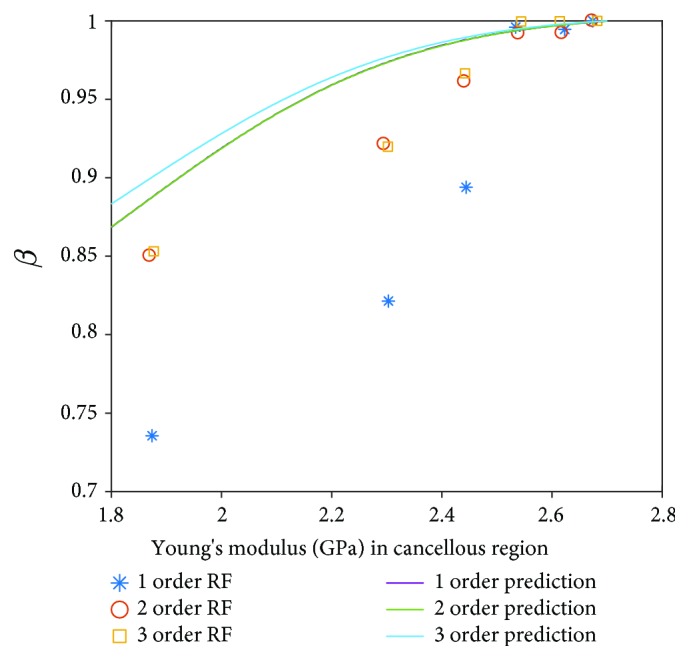
Comparison between deterministic results and prediction results of RF in the cancellous region.

In order to have quantitative comparison, the prediction results of the Kriging surrogate model and the results in the literature [36] are presented in Figures 10 and 11. In clinical and experimental observation, RF increases and changes in the beginning of the first several months and reaches a certain steady state after a period. In the healing process, the Young's modulus of the cortical region and cancellous region increases. Therefore, the other input variables are settled as certain values in the Kriging surrogate model; the Young's modulus of cortical (E3) and cancellous (E4) bones are input variables.

From Figure 10, it can be found that in general, according to the increase of the Young's modulus cortical region, the first three order RFs are all amplified in the beginning period in the results of the literature [36] and the prediction of the Kriging surrogate model, where *β* is the ratio of the RF related to the specific Young's modulus with the steady RF in the final phase. Due to the database of LH-FEM, the first-order and the second-order prediction results are very close, while the prediction results of the Kriging surrogate model in the third RF are approximated with the results in the work of Glauser et al. [26], especially in the beginning and end parts of the result curve.

Furthermore, the prediction results of RF in the cancellous region by the Kriging surrogate model are also compared with the results in the literature [36] as shown in Figure 11. A good agreement is achieved when the Young's modulus in the cancellous region is large. However, the prediction results of the Kriging surrogate model are larger than the deterministic results [36] when the Young's modulus in the cancellous region is large. The reasons causing this phenomenon can be the original database of LH-FEM, the low sensitivity of the Kriging surrogate model for the cancellous region, or computational relative errors of the method applied in this study. On the other hand, the Kriging surrogate model provides continuous result prediction of RF for the dental implant system, which is more convenient, comprehensive, and time-saving for RFA than the traditional finite element method calculation and clinical test.

In addition, based on the accuracy and reliability of the Kriging surrogate model prediction, it is also useful in the analysis process of dental implant stability. However, the dental stability cannot be directly measured or simulated with an in vivo or vitro model under specific clinical situations. The Kriging surrogate model proposed in this study can play important roles and provide believable prediction results of RF corresponding with the dental stability. Besides, the Kriging surrogate model is compatible to combine the numerical simulation results with clinical and experimental results in the original database, which makes it a more sophisticated surrogate model for the dental implant system.

Through LH-FEM proposed in this paper, a series of meaningful results are obtained for RFA of dental implants. However, there are still some limitations caused by simplification and assumptions involving in the numerical simulation. Firstly, material properties of each component in the dental implant system are supposed to be homogenous and isotropic. Secondly, the damping effect is totally neglected during the RFA, while damping may have a certain extent influence on the accuracy of RF. Thirdly, the interfaces in the system are all treated as perfect bonding without considering the local specialty. In the real operation situation, the bone-implant interface is a dynamic living surface that evolves from a debonded interface to a bonded interface. Li et al. [60] applied the bonded model to calculate the RF of the bone-implant interface in a dental implant. The results were in a quantitative agreement with some experimental measurements, because the bone-implant interface was fully osseointegrated after several weeks [4, 61]. Despite such simplification in the simulation process, the computational results in RFA are fairly reasonable, which could be useful and helpful for implant designers and prosthetic researchers. Furthermore, the prediction results of the Kriging surrogate model for RFA of dental implants by LH-FEM are reliable and believable.

## 4. Conclusion

In conclusion, the computational model proposed in this paper is a successful numerical tool for noninvasive RFA in dental implant research. LH-FEM is appropriate and feasible to study the influence of the material properties of the implant medical components on RF. The Kriging surrogate model is an effective model to precisely describe the relationship between RF and parameters of material property. The output results of RF from LH-FEM are in the reasonable and believable range. The prediction results from the Kriging surrogate model have a good agreement with the published paper. Based on the database of LH-FEM, the Kriging surrogate model is an appropriate and powerful method in RFA of dental implants.

## Figures and Tables

**Figure 12:**
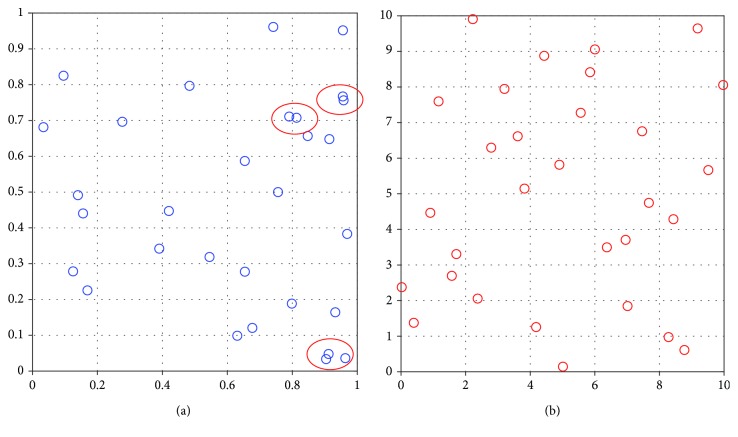
Comparison between the LH sampling method and MCS ((a) for MCS, (b) for the LH sampling method).

**Table 1:** Parameters of geometrical properties in FEM.

	Definition	mm
H1	The height of the bottom part in dental implants	14.2
H2	The height of the middle part in dental implants	2
H3	The height of the top part in dental implants	5
*H*	The height of the cortical bone	26
h1	The height (in the front end) of the thread in dental implants	0.1
*h*	The distance (in the front end) between the edges of the thread in dental implants	0.9
*W*	The width of whole FEM	16
W1	The thickness of the cortical bone	1.3
W2	The diameter of the ceramic crown	5.1
W3	The diameter of dental implants	3.1
*w*	The width of teeth in the thread part of dental implants	0.4
H4	The height of the ceramic crown	2

Besides, *d* represents the degree of the included angle in the front end of thread and is settled as50°.

**Table 2:** Physical properties of materials for FEM.

Material	Young's modulus range (GPa)	Poisson's ratio range	Physical density (kg/m^3^)
Dental implant	E1 (100–200)	R1 (0.25–0.35)	D1 (4000–8000)
Ceramic crown	E2 (5–12)	R2 (0.25–0.35)	D2 (2000–3000)
Cortical bone	E3 (10.0–20.0)	R3 (0.25–0.35)	D3 (1600–2000)
Cancellous bone	E4 (0.8–1.5)	R4 (0.25–0.35)	D4 (1600–2000)

**Table 3:** Statistical results of LH-FEM for RFA.

	Maximum (kHz)	Minimum (kHz)	Mean (kHz)	Variance (kHz)
F1	10.0	6.1	7.8	0.7
F2	10.1	6.1	7.9	0.7
F3	28.2	17.2	21.9	4.4
F4	35.7	21.5	27.9	7.9
F5	37.0	22.4	28.8	8.3

## Data Availability

The original data used to support the findings of this study are available from the corresponding author upon request.

## Appendix

### A.1. Kriging Surrogate Model

A surrogate model likes a black box, where the mathematical relationship between the parameters in the input database and parameters in the output results is expressed by approximated implicit methods. Usually, the surrogate model can be classified into two kinds of groups: local and global models. The response surface method is a typical local model and can be written in polynomial series as follows [49]:

(A.1) $$\begin{array}{c} F\left(\beta :x\right)=\beta_{0}+\overset{n}{\underset{i=1}{\sum }}\beta_{i}x_{i}, \end{array}$$

(A.2) $$\begin{array}{c} F\left(\beta :x\right)=\beta_{0}+\overset{n}{\underset{i=1}{\sum }}\beta_{i}x_{i}+\overset{n}{\underset{i=1}{\sum }}\overset{n}{\underset{j=1}{\sum }}\beta_{ij}x_{i}x_{j}. \end{array}$$

Equation (A.1) and equation (A.2) are the local first- and second-order response surface model, respectively, where *F*(*β* : *x*) is a deterministic regression model and *β*_*i*_ is the corresponding regression coefficient.

In addition, a global surrogate model generally has global searching space. Kriging models fit a spatial correlation function as follows:

(A.3) $$\begin{array}{c} G\left(x\right)=F\left(\beta :x\right)+z\left(x\right), \end{array}$$

where *z*(*x*) is assumed to have mean zero and covariance.

In the first-order linear Kriging surrogate model, the predictor can be expressed as follows [63]:

(A.4) $$\begin{array}{c} \hat{y}\left(x\right)=c^{T}Y, \end{array}$$

where *c* = *c*(*x*) ∈ *R*^*m*^. The relative error is

(A.5) $$\begin{array}{c} \hat{y}\left(x\right)-y\left(x\right)=c^{T}Y-y\left(x\right)=c^{T}\left(F\beta +Z\right)-\left(f{\left(x\right)}^{T}\beta +z\right)=c^{T}Z-z+{\left(F^{T}c-f\left(x\right)\right)}^{T}\beta . \end{array}$$

In order to make sure the predictor is unbiased, *F*^*T*^*c* is equal to *f*(*x*). The mean squared error of the predictor can be written as follows:

(A.6) $$\begin{array}{c} \varphi \left(x\right)=E\left[{\left(\overset{\wedge }{y}\left(x\right)-y\left(x\right)\right)}^{2}\right]=E\left[{\left(c^{T}Z-z\right)}^{2}\right]=\sigma^{2}\left(1+c^{T}Rc-2c^{T}r\right). \end{array}$$

The objective of the optimization program is to have the minimum value of *φ*; the constraint is

(A.7) $$\begin{array}{c} L\left(c,\lambda \right)=\sigma^{2}\left(1+c^{T}Rc-2c^{T}r\right)-\lambda^{T}\left(F^{T}c-f\right). \end{array}$$

The computation of the gradient of the constraint function with respect to *c* can be expressed as follows:

(A.8) $$\begin{array}{c} L_{c}^{\prime }\left(c,\lambda \right)=2\sigma^{2}\left(Rc-r\right)-F\lambda . \end{array}$$

Suppose $\tilde{\lambda }=-\lambda /2\sigma^{2}$, the following system of equations is

(A.9) $$\begin{array}{c} \left[\begin{array}{cc} R & F\\ F^{T} & 0 \end{array}\right]\left[\begin{array}{c} c\\ \tilde{\lambda } \end{array}\right]=\left[\begin{array}{c} r\\ f \end{array}\right]. \end{array}$$

The solution can be obtained:

(A.10) $$\begin{array}{c} \tilde{\lambda }={\left(F^{T}R^{-1}F\right)}^{-1}\left(F^{T}R^{-1}r-f\right),\\ c=R^{-1}\left(r-F\tilde{\lambda }\right), \end{array}$$

where *R*^−1^ is the inverse matrix of the correlation matrix *R*.

(A.11) $$\begin{array}{c} \hat{y}\left(x\right)={\left(r-F\tilde{\lambda }\right)}^{T}R^{-1}Y=r^{T}R^{-1}Y-{\left(F^{T}R^{-1}r-f\right)}^{T}{\left(F^{T}R^{-1}F\right)}^{-1}F^{T}R^{-1}Y. \end{array}$$

The generalized least squares solution is

(A.12) $$\begin{array}{c} \beta^{*}={\left(F^{T}R^{-1}F\right)}^{-1}F^{T}R^{-1}Y. \end{array}$$

Substitute *β*^∗^ into the predictor of the Kriging surrogate model,

(A.13) $$\begin{array}{c} \hat{y}\left(x\right)=r^{T}R^{-1}Y-{\left(F^{T}R^{-1}r-f\right)}^{T}\beta^{*}=f^{T}\beta^{*}+r^{T}R^{-1}\left(Y-F\beta^{*}\right)=f{\left(x\right)}^{T}\beta^{*}+r{\left(x\right)}^{T}\gamma^{*}. \end{array}$$

Besides, the corresponding maximum likelihood estimate of the variance is written as follows:

(A.14) $$\begin{array}{c} \sigma^{2}=\frac{1}{m}{\left(Y-F\beta^{*}\right)}^{T}\left(Y-F\beta^{*}\right). \end{array}$$

If the errors are uncorrelated and have different variances, E[*e*_*i*_*e*_*i*_] = *σ*_*i*_^2^ and E[*e*_*i*_*e*_*j*_] = 0 for i ≠ j. It is logical to find that *R* is the diagonal matrix,

(A.15) $$\begin{array}{c} R=\mathrm{diag}\left(\frac{\sigma_{1}^{2}}{\sigma^{2}},\cdots ,\frac{\sigma_{m}^{2}}{\sigma^{2}}\right). \end{array}$$

Besides, the weight matrix *W* is given as follows:

(A.16) $$\begin{array}{c} W=\mathrm{diag}\left(\frac{\sigma }{\sigma_{1}},\ldots ,\frac{\sigma }{\sigma_{m}}\right)\Leftrightarrow W^{2}=R^{-1}. \end{array}$$

Therefore,

(A.17) $$\begin{array}{c} \tilde{Y}=WY=WF\beta +\tilde{e}. \end{array}$$

And, the below equations are satisfied:

(A.18) $$\begin{array}{c} E\left[\tilde{e}\right]=0,\\ E\left[{\tilde{e}\tilde{e}}^{T}\right]=\text{WE}\left[{ee}^{T}\right]W^{T}=\sigma^{2}I. \end{array}$$

Replacing *F* and *Y* by weighted function, the results can be depicted as follows:

(A.19) $$\begin{array}{c} \left(F^{T}W^{2}F\right)\beta^{*}=F^{T}W^{2}Y,\\ \;\sigma^{2}=\frac{1}{m}{\left(Y-F\beta^{*}\right)}^{T}W^{2}\left(Y-F\beta^{*}\right). \end{array}$$

### A.2. Resonance Frequency

Modal analysis is efficient to identify the RF of objects in the fields of engineering, industry, and medicine. Resonance happens when a structural system vibrates at its RF with a tendency to oscillate in higher amplitudes. Since the value of RF is associated with material stiffness, structural damping, physical density, and boundary condition, RF can be employed to symbolize a structural system.

The calculation of RF of a system is essentially a generalized eigenvalue problem. The free vibration without damping is governed by the following equation:

(A.20) $$\begin{array}{c} \left[M\right]\left\{\ddot{u}\right\}+\left[K\right]\left\{u\right\}=\left\{0\right\}, \end{array}$$

where [*K*] is the structure stiffness matrix and [*M*] is the structure mass matrix.

For the linear system, free vibrations will be harmonic of the form:

(A.21) $$\begin{array}{c} \left\{u\right\}=\left\{\phi_{i}\right\}\cos\omega_{i}t. \end{array}$$

In the above equation, {*ϕ*_*i*_} is eigenvector representing the mode shape of the *i*th natural frequency, *ω*_*i*_ is the *i*th natural circular frequency, and *t* is time.

Thus, the free vibration can be presented as follows:

(A.22) $$\begin{array}{c} \left(-\omega_{i}^{2}\left[M\right]+\left[K\right]\right)\left\{\phi_{i}\right\}=\left\{0\right\}. \end{array}$$

This equality is satisfied if either {*ϕ*_*i*_} = {0} or (−*ω*_*i*_^2^[*M*] + [*K*]) equals to zero. Then,

(A.23) $$\begin{array}{c} \left|\left[K\right]-\omega^{2}\left[M\right]\right|=0. \end{array}$$

This is an eigenvalue problem which may be solved for up to *n* values of *ω*^2^ and *n* eigenvectors {*ϕ*_*i*_}, where *n* is the number of degree of freedom.

The eigenvalue and eigenvector problem needs to be solved for mode-frequency and buckling analyses. It also has the form as follows:

(A.24) $$\begin{array}{c} \left[K\right]\left\{\phi_{i}\right\}=\lambda_{i}\left[M\right]\left\{\phi_{i}\right\}, \end{array}$$

where *λ*_*i*_ is an eigenvalue. The block Lanczos method uses the sparse direct solver to perform Lanczos iterations and extracts the requested eigenvalues.

Rather than the natural circular frequencies {*ω*}, the natural frequency (*f*) is

(A.25) $$\begin{array}{c} f_{i}=\frac{\omega_{i}}{2\pi }. \end{array}$$

Therefore, the natural frequency (*f*) is not only corresponding with structure stiffness matrix [*K*] but also be affected by [*M*] structure mass matrix. It is an effective index to observe the changes or deviation of the structure geometry and material property.

### A.3. Latin Hypercube Sampling Method

The Latin Hypercube sampling method is one kind of advanced Monte Carlo simulation (MCS). This approach divides the range of each variable into disjoint intervals of equal probability, and one value is randomly selected from each interval. It improves the stability of MCS and keeps satisfied accuracy and good convergence [49, 50]. Consider a statistic system described by the function,

(A.26) $$\begin{array}{c} Y=F\left(X\right),\;X=\left\{X_{1},X_{2},\cdots ,X_{n}\right\}, \end{array}$$

where *X* is the random vector and represents the independent input random variables. *F* is the operator and performs computer simulation, such as finite element computation.

Traditional MCS relies on simple random sampling, in which realization of *X*, denoted by *x*_*k*_, *k* = 1,…, *N*, where *N* is the amount of samples. In the sample space,

(A.27) $$\begin{array}{c} x_{ki}=P_{X_{i}}^{-1}\left(U_{i}\right),\;i=1,\ldots ,n, \end{array}$$

where *U*_*i*_ are the uniformly distributed samples on [0, 1] and *P* is the cumulative distribution function. Besides, Nataf or Rosenblatt transformations can be used to produce a set of uncorrelated random variables from correlated variables.

Latin Hypercube sampling method divides the range of each vector component into disjoint subsets of equal probability. Samples of each vector component are captured from the respective subsets according to equation as follows:

(A.28) $$\begin{array}{c} x_{ki}^{j}=P_{X_{i}}^{-1}\left(U_{ij}\right), \end{array}$$

where *i* = 1,…, *n* and *j* = 1,…, *m*, where *n* refers to the total number of vector components or dimensions of the vector and *m* is the number of the subset in a design. *k* is the subscript which denotes a specific sample.

Besides, *U*_*ij*_ are uniformly distributed samples on [*ξ*_*j*_, *ξ*_*j*_′],

(A.29) $$\begin{array}{c} \xi_{j}=\frac{j-1}{m},\\ \xi_{j}^{\prime }=\frac{j}{m},\\ x_{ki}^{j}=P_{X_{i}}^{-1}\left(U_{ij}\right). \end{array}$$

By the Latin Hypercube sampling method, the ranges of all random input variables are all divided into intervals with equal probability, the efficiency of sampling is improved, and the disadvantage of clustering points is well overcome, as presented in Figure 12.

In Figure 12, the data are the examples to compare the efficiency of the Latin Hypercube sampling method and Monte Carlo method. Actually, it can be seen as the example of sampling space of parameters in the model of dental implants.

## References

[1] Capek L., Simunek A., Slezak R., Dzan L. (2009). Influence of the orientation of the Osstell® transducer during measurement of dental implant stability using resonance frequency analysis: a numerical approach. *Medical Engineering and Physics*.

[2] Wang S., Liu G. R., Hoang K. C., Guo Y. (2010). Identifiable range of osseointegration of dental implants through resonance frequency analysis. *Medical Engineering and Physics*.

[3] Genna F. (2003). On the effects of cyclic transversal forces on osseointegrated dental implants: experimental and finite element shakedown analyses. *Computer Methods in Biomechanics and Biomedical Engineering*.

[4] Pérez M. A., Moreo P., García-Aznar J. M., Doblaré M. (2008). Computational simulation of dental implant osseointegration through resonance frequency analysis. *Journal of Biomechanics*.

[5] Rungsiyakull C., Li Q., Sun G., Li W., Swain M. V. (2010). Surface morphology optimization for osseointegration of coated implants. *Biomaterials*.

[6] Schulte W., Lukas D. (1993). Periotest to monitor osseointegration and to check the occlusion in oral implantology. *The Journal of Oral Implantology*.

[7] Bischof M., Nedir R., Szmukler-Moncler S., Bernard J. P., Samson J. (2004). Implant stability measurement of delayed and immediately loaded implants during healing. *Clinical Oral Implants Research*.

[8] Miyamoto I., Tsuboi Y., Wada E., Suwa H., Iizuka T. (2005). Influence of cortical bone thickness and implant length on implant stability at the time of surgery—clinical, prospective, biomechanical, and imaging study. *Bone*.

[9] Hsu J. T., Shen Y. W., Kuo C. W., Wang R. T., Fuh L. J., Huang H. L. (2017). Impacts of 3D bone-to-implant contact and implant diameter on primary stability of dental implant. *Journal of the Formosan Medical Association*.

[10] Atsumi M., Park S. H., Wang H. L. (2007). Methods used to assess implant stability: current status. *International Journal of Oral & Maxillofacial Implants*.

[11] Nkenke E., Hahn M., Weinzierl K., Radespiel-Tröger M., Neukam F. W., Engelke K. (2003). Implant stability and histomorphometry: a correlation study in human cadavers using stepped cylinder implants. *Clinical Oral Implants Research*.

[12] Meredith N., Alleyne D., Cawley P. (1996). Quantitative determination of the stability of the implant-tissue interface using resonance frequency analysis. *Clinical Oral Implants Research*.

[13] Wirth A. J., Goldhahn J., Flaig C., Arbenz P., Müller R., van Lenthe G. H. (2011). Implant stability is affected by local bone microstructural quality. *Bone*.

[14] Aparicio C., Lang N. P., Rangert B. (2006). Validity and clinical significance of biomechanical testing of implant/bone interface. *Clinical Oral Implants Research*.

[15] Kim D. S., Lee W. J., Choi S. C. (2014). Comparison of dental implant stabilities by impact response and resonance frequencies using artificial bone. *Medical Engineering and Physics*.

[16] Lin D., Li Q., Li W., Swain M. (2010). Bone remodeling induced by dental implants of functionally graded materials. *Journal of Biomedical Materials Research Part B: Applied Biomaterials*.

[17] Huwiler M. A., Pjetursson B. E., Bosshardt D. D., Salvi G. E., Lang N. P. (2007). Resonance frequency analysis in relation to jawbone characteristics and during early healing of implant installation. *Clinical Oral Implants Research*.

[18] Ito Y., Sato D., Yoneda S., Ito D., Kondo H., Kasugai S. (2008). Relevance of resonance frequency analysis to evaluate dental implant stability: simulation and histomorphometrical animal experiments. *Clinical Oral Implants Research*.

[19] Lachmann S., Jäger B., Axmann D., Gomez-Roman G., Groten M., Weber H. (2006). Resonance frequency analysis and damping capacity assessment. Part 1: an *in vitro* study on measurement reliability and a method of comparison in the determination of primary dental implant stability. *Clinical Oral Implants Research*.

[20] Lachmann S., Yves Laval J., Jäger B. (2006). Resonance frequency analysis and damping capacity assessment. Part 2: peri-implant bone loss follow-up. An in vitro study with the Periotest and Osstell instruments. *Clinical Oral Implants Research*.

[21] Huang H. M., Pan L. C., Lee S. Y., Chiu C. L., Fan K. H., Ho K. N. (2000). Assessing the implant/bone interface by using natural frequency analysis. *Oral Surgery, Oral Medicine, Oral Pathology, Oral Radiology, and Endodontics*.

[22] Pattijn V., van Lierde C., van der Perre G., Naert I., Vander Sloten J. (2006). The resonance frequencies and mode shapes of dental implants: rigid body behaviour versus bending behaviour. A numerical approach. *Journal of Biomechanics*.

[23] Harirforoush R., Arzanpour S., Chehroudi B. (2014). The effects of implant angulation on the resonance frequency of a dental implant. *Medical Engineering & Physics*.

[24] Tözüm T. F., Turkyilmaz I., Yamalik N., Karabulut E., Eratalay K. (2007). Analysis of the potential association of implant stability, laboratory, and image-based measures used to assess osteotomy sites: early versus delayed loading. *Journal of Periodontology*.

[25] Schliephake H., Sewing A., Aref A. (2006). Resonance frequency measurements of implant stability in the dog mandible: experimental comparison with histomorphometric data. *International Journal of Oral and Maxillofacial Surgery*.

[26] Glauser R., Sennerby L., Meredith N. (2004). Resonance frequency analysis of implants subjected to immediate or early functional occlusal loading. Successful vs. failing implants. *Clinical Oral Implants Research*.

[27] Chou H. Y., Jagodnik J. J., Müftü S. (2008). Predictions of bone remodeling around dental implant systems. *Journal of Biomechanics*.

[28] Li J., Li H., Shi L. (2007). A mathematical model for simulating the bone remodeling process under mechanical stimulus. *Dental Materials*.

[29] Lin D., Li Q., Li W., Zhou S., Swain M. V. (2009). Design optimization of functionally graded dental implant for bone remodeling. *Composites Part B: Engineering*.

[30] Aksoy U., Eratalay K., Tözüm T. F. (2009). The possible association among bone density values, resonance frequency measurements, tactile sense, and histomorphometric evaluations of dental implant osteotomy sites: a preliminary study. *Implant Dentistry*.

[31] Nakatsuchi Y., Tsuchikane A., Nomura A. (1996). The vibrational mode of the tibia and assessment of bone union in experimental fracture healing using the impulse response method. *Medical Engineering & Physics*.

[32] Iida T., Mukohyama H., Inoue T. (2001). Modal analysis of the maxillary dentition in cleft lip and palate patients before and after bone grafting. *Journal of Medical and Dental Sciences*.

[33] Natali A. N., Pavan P. G., Schileo E., Williams K. R. (2006). A numerical approach to resonance frequency analysis for the investigation of oral implant osseointegration. *Journal of Oral Rehabilitation*.

[34] De Smet E., Jaecques S. V., Jansen J. J., Walboomers F., Vander Sloten J., Naert I. E. (2008). Effect of strain at low-frequency loading on peri-implant bone (re) modelling: a guinea-pig experimental study. *Clinical Oral Implants Research*.

[35] Deng B., Tan K. B., Liu G. R., Lu Y. (2008). Influence of osseointegration degree and pattern on resonance frequency in the assessment of dental implant stability using finite element analysis. *International Journal of Oral & Maxillofacial Implants*.

[36] Li W., Lin D., Rungsiyakull C., Zhou S., Swain M., Li Q. (2011). Finite element based bone remodeling and resonance frequency analysis for osseointegration assessment of dental implants. *Finite Elements in Analysis and Design*.

[37] Pérez M. A. (2012). Life prediction of different commercial dental implants as influence by uncertainties in their fatigue material properties and loading conditions. *Computer Methods and Programs in Biomedicine*.

[38] Burrage K., Burrage P., Donovan D., Thompson B. (2015). Populations of models, experimental designs and coverage of parameter space by Latin hypercube and orthogonal sampling. *Procedia Computer Science*.

[39] Cioppa T. M., Lucas T. W. (2007). Efficient nearly orthogonal and space-filling Latin hypercubes. *Technometrics*.

[40] Joseph V. R., Hung Y. (2008). Orthogonal-maximin Latin hypercube designs. *Statistica Sinica*.

[41] Zhang P., Breitkopf P., Knopf-Lenoir C., Zhang W. (2011). Diffuse response surface model based on moving Latin hypercube patterns for reliability-based design optimization of ultrahigh strength steel NC milling parameters. *Structural and Multidisciplinary Optimization*.

[42] Liefvendahl M., Stocki R. (2006). A study on algorithms for optimization of Latin hypercubes. *Journal of Statistical Planning and Inference*.

[43] Shields M. D., Zhang J. (2016). The generalization of Latin hypercube sampling. *Reliability Engineering & System Safety*.

[44] Park J. S. (1994). Optimal Latin-hypercube designs for computer experiments. *Journal of Statistical Planning and Inference*.

[45] Vořechovský M., Novák D. (2009). Correlation control in small-sample Monte Carlo type simulations I: a simulated annealing approach. *Probabilistic Engineering Mechanics*.

[46] Stocki R. (2005). A method to improve design reliability using optimal Latin hypercube sampling. *Computer Assisted Mechanics and Engineering Sciences*.

[47] Ghiocel D. M., Ghanem R. G. (2002). Stochastic finite-element analysis of seismic soil–structure interaction. *Journal of Engineering Mechanics*.

[48] Shu Z., Jirutitijaroen P. (2011). Latin hypercube sampling techniques for power systems reliability analysis with renewable energy sources. *IEEE Transactions on Power Systems*.

[49] Chu L., De Cursi E. S., El Hami A., Eid M. (2015). Application of Latin hypercube sampling based kriging surrogate models in reliability assessment. *Science Journal of Applied Mathematics and Statistics*.

[50] Chu L., De Cursi E. S., El Hami A., Eid M. (2015). Reliability based optimization with metaheuristic algorithms and Latin hypercube sampling based surrogate models. *Applied and Computational Mathematics*.

[51] Sheikholeslami R., Razavi S. (2017). Progressive Latin hypercube sampling: an efficient approach for robust sampling-based analysis of environmental models. *Environmental Modelling & Software*.

[52] Matheron G. (1963). Principles of geostatistics. *Economic Geology*.

[53] Lelièvre N., Beaurepaire P., Mattrand C., Gayton N. (2018). AK-MCSi: a Kriging-based method to deal with small failure probabilities and time-consuming models. *Structural Safety*.

[54] Rennen G. (2009). Subset selection from large datasets for kriging modeling. *Structural and Multidisciplinary Optimization*.

[55] Pistone G., Vicario G. (2010). Comparing and generating Latin hypercube designs in Kriging models. *AStA Advances in Statistical Analysis*.

[56] Sen O., Gaul N. J., Choi K. K., Jacobs G., Udaykumar H. S. (2017). Evaluation of kriging based surrogate models constructed from mesoscale computations of shock interaction with particles. *Journal of Computational Physics*.

[57] Dubreuil S., Bartoli N., Gogu C., Lefebvre T., Colomer J. M. (2018). Extreme value oriented random field discretization based on an hybrid polynomial chaos expansion — Kriging approach. *Computer Methods in Applied Mechanics and Engineering*.

[58] Cousin A., Maatouk H., Rullière D. (2016). Kriging of financial term-structures. *European Journal of Operational Research*.

[59] Simpson T. W., Poplinski J. D., Koch P. N., Allen J. K. (2001). Metamodels for computer-based engineering design: survey and recommendations. *Engineering With Computers*.

[60] Li Y. F., Ng S. H., Xie M., Goh T. N. (2010). A systematic comparison of metamodeling techniques for simulation optimization in decision support systems. *Applied Soft Computing*.

[61] Huang H. M., Cheng K. Y., Chen C. F., Ou K. L., Lin C. T., Lee S. Y. (2005). Design of a stability-detecting device for dental implants. *Proceedings of the Institution of Mechanical Engineers, Part H: Journal of Engineering in Medicine*.

[62] Flanagan D., Ilies H., McCullough P., McQuoid S. (2008). Measurement of the fatigue life of mini dental implants: a pilot study. *Journal of Oral Implantology*.

[63] Lophaven S. N., Nielsen H. B., Sondergaard J. (2002). *DACE-A MATLAB Kriging Toolbox*.

